# Pancreatic beta-cell secretory products in the diagnosis and risk stratification of gestational diabetes mellitus: a prospective longitudinal cohort study

**DOI:** 10.1136/bmjopen-2025-100039

**Published:** 2025-09-05

**Authors:** Gareth Dunseath, Michael Atkinson, Wai-Yee Cheung, Stephen Luzio, Rajesh Peter

**Affiliations:** 1 Diabetes Research Group, Swansea University Faculty of Medicine Health and Life Science, Swansea, Wales, UK; 2 Neath Port Talbot Hospital, Port Talbot, Wales, UK

**Keywords:** Diabetes in pregnancy, Pregnancy, Diabetes & endocrinology

## Abstract

**Introduction:**

Gestational diabetes mellitus (GDM) is common in pregnancy and is increasing in prevalence. It is associated with an increased risk of maternal and perinatal complications if not diagnosed and managed early. Most guidelines suggest making a diagnosis of GDM using an oral glucose tolerance test (OGTT) between 24 and 28 weeks of pregnancy at which stage there still is an increased risk of complications. Increased beta-cell secretory product concentrations have been observed prior to changes in glycaemia and can potentially be used as an early marker to diagnose and assess risk of developing GDM.

**Methods:**

The study was a prospective, longitudinal cohort study. OGTTs were carried out at visit one: 16–18 weeks and visit two: 24–28 weeks gestation in pregnant women with at least one risk factor for GDM [Body Mass Index >30 kg/m^2^, previous macrosomic baby (>4.5 kg), previous GDM, first degree relative with type 2 diabetes mellitus (T2DM)]. Blood sampling was performed at fasting, 30 min, 1 and 2 hours following a 75-g oral glucose load. Samples were analysed for glucose, total and intact proinsulin, insulin and C-peptide. Hormonal concentrations at visit 1 were compared between those that remained normal glucose tolerant (NGT) and those that progressed to GDM at visit 2 using receiver operator characteristic (ROC) area under the curve (AUC) to assess for discrimination between the two groups.

**Results:**

Unfortunately, a smaller than planned sample size was recruited due to the start of COVID-19 pandemic midway through the study. 83 pregnant women had OGTT at visit 1. Of these, 12 reached the threshold for GDM at visit 1 and were excluded. In total, data from 66 patients were included for analysis (5 Did Not Attend). Visit 1 hormone comparisons were carried out between 51 who remained NGT and 15 who progressed to GDM at visit 2. There were no significant differences at each time point in ROC AUC between the two groups for total and intact proinsulin and insulin. However, there were significant differences observed in C-peptide ROC AUC at 30 (p=0.041) and 60 min (p=0.003) between the two groups.

**Conclusions:**

This study did not demonstrate significant increase in early proinsulin concentrations in patients that developed GDM. However, there were differences in C-peptide concentrations. The COVID-19 pandemic restricted the recruitment of patient numbers and further studies in a larger cohort will be needed to validate these findings.

**Trial registration number:**

ISRCTN16416602.

**STRENGTHS AND LIMITATIONS OF THIS STUDY**

- This was a prospective, longitudinal cohort study recruiting at a single site.
- Proinsulin and C-peptide were measured using sensitive and highly specific immunoassays, eliminating the cross-reactivity of other beta-cell products observed with many other immunoassays.
- A smaller than planned sample size was recruited due to the restrictions imposed by the COVID-19 pandemic; findings therefore need to be interpreted with care.

## Introduction

 It is estimated that hyperglycaemia in pregnancy affects approximately one in five pregnancies and 75%–90% of these are due to gestational diabetes mellitus (GDM).^1^ Within England and Wales, approximately 35 000 pregnancies were affected with hyperglycaemia, of which 87.5% had GDM.^2^ As most women with GDM are asymptomatic and the condition is associated with an increased risk of maternal and perinatal complications, it is imperative that high-risk women are screened in a timely, accurate and reliable way. Diagnosis of GDM is made in the late second trimester or early third trimester and in the UK, women with a high risk of GDM (Body Mass Index (BMI) >30 kg/m^2^; previous macrosomic baby >4.5 kg; previous GDM; first degree relative with type 2 diabetes mellitus (T2DM) and certain ethnicities, eg, south Asian) are offered a 75-g oral glucose tolerance test (OGTT) at 24–28 weeks gestation.^2^

Glycated haemoglobin (HbA_1c_)cannot reliably be used to diagnose GDM as there is increased red cell turnover in pregnancy. A systematic review of 10 cohort studies (n=16 254), in which HbA_1c_ testing was undertaken to detect early GDM (at least 6 of the studies specified that testing had taken place prior to 20 weeks gestation) demonstrated that the risk of developing GDM increases when HbA_1c_ is ≥39 mmol/mol and a HbA_1c_ concentration ≥42 mmol/mol identifies most patients who go on to develop GDM.^3^ Changes to diagnosis of GDM during the COVID-19 pandemic were forced on maternity services in the UK with the use of either HbA_1c_ ≥39 mmol/mol or a single glucose sample (either fasting ≥5.3 mmol/L or random glucose sample ≥9 mmol/L).^4^ A retrospective study of prospectively collected data^5^ demonstrated that using the Royal College of Obstetricians and Gynaecologists (RCOG) COVID-19 gestational diabetes screening guideline failed to detect 47 of the 82 (57%) women subsequently identified with GDM and hence cannot reliably be recommended for use.

A scoping review of the guidelines and diagnostic studies evaluating the recommended testing strategies concluded that OGTT remains the most effective test which resulted in the return of standard guidelines post-pandemic.^6^ National Institute for Health and Care Excellence (NICE) guidelines (2015) still recommend risk factor screening optimally at 24–28 weeks using a 75-g oral glucose load with fasting plasma glucose ≥5.6 mmol/L, or 2-hour plasma glucose ≥7.8 mmol/L to diagnose GDM.^2^

A number of potential biomarkers for early detection of GDM have been studied. There is some suggestion that leptin levels are higher in women with GDM, although this is confounded by ovarian and placenta leptin production.^7^ Adiponectin is not produced by the ovaries nor placenta, and one study has shown lower adiponectin levels during the first and second trimester in women who go on to develop GDM in the third trimester.^7^ Associations between future GDM and sex hormone binding globulin^8^ and uric acid levels^9^ have also been suggested. A previous study has shown that in pregnant women with obesity, measurement of fructosamine, adiponectin, sex hormone binding globulin and triglycerides, in addition to HbA_1c_ and random glucose, combined with other variables (age, previous GDM, family history of type 2 diabetes, systolic blood pressure, sum of skinfold thicknesses, waist:height, and neck:thigh ratios) achieved an area under the receiver operator characteristics curve (AUC) of 0.77 (95% CI 0.73 to 0.80).^10^

Although studies have identified a range of amino acid and other biochemical markers, it has not been possible to draw any firm conclusions. These include afamin,^11^ angiopoietin-like protein 8,^12^ extracellular vesicles,^13^ Fibroblast Growth Factor 21 (FGF21),^14^ placental growth factor and follistatin-like 3,^15^ and pregnancy-associated plasma protein-A.^16^ Additional studies have looked at visfatin and human fetuin A^17 18^ and plasma glycated CD59 (pGCD59)^19^ as potential biomarkers.

Pregnancy is initially associated with a decrease in insulin resistance which promotes glucose uptake in preparation for the rapid fetus growth phase in late pregnancy.^20^ Fasting plasma glucose levels are therefore usually lower in early pregnancy compared with the non-pregnant state.^21^ As pregnancy progresses, there is a gradual increase in insulin resistance through the second and third trimesters which peaks in late gestation at approximately 150%–160% of the prepregnancy state.^22^ To counteract this resistance, pancreatic beta cells secrete more insulin as gestation progresses to maintain glycaemic control as is also seen in people with T2DM.^23^ However, if and when pancreatic beta cells cannot produce sufficient insulin to counteract this physiological effect, hyperglycaemia results. Also, insulin-stimulated glucose uptake is 50%–60% lower in GDM when compared with non-GDM pregnancies.^23 24^

Proinsulin is a precursor molecule for insulin and is synthesised by pancreatic beta cells. Under normal circumstances, almost all proinsulin is cleaved to insulin and C-peptide with a small amount of intact proinsulin along with split proinsulin also released. When insulin resistance rises, pancreatic beta-cells go into overdrive with resultant increased secretion of not just insulin and C-peptide but also disproportionately more proinsulin (both intact and split) as is seen in people with T2DM.^25^

Previous studies in non-pregnant women and those with GDM have demonstrated elevated fasting proinsulin concentrations in GDM.^26 27^ These studies, however, looked at women already diagnosed with GDM at 24–28 weeks and did not assess the use of proinsulin as a screening biomarker. Moreover, studies assessing proinsulin in pregnancy did not take into account individual risk factors which may account for varied findings.^28 29^ Therefore, we hypothesised that plasma proinsulin with its breakdown products (insulin and C-peptide) may potentially be used as early screening biomarkers to identify pregnant women with risk factors who will go on to develop GDM.

The original primary aim of the study was to establish if proinsulin concentrations at 16–18 weeks gestation would help identify or risk stratify high-risk pregnant women who go on to develop GDM according to NICE criteria.^2^ However, due to the early termination of the study, the primary aim was amended to *explore* whether proinsulin concentrations at 16–18 weeks would help identify or risk stratify high-risk pregnant women who go on to develop GDM according to NICE criteria. We also measured insulin and C-peptide and looked to establish if fasting and post-oral glucose concentrations can discriminate women who will develop GDM.

## Methods

This was a prospective, longitudinal cohort study, carried out between November 2017 and January 2020. The study was approved by the Wales Research Ethics Committee (Panel 6, REC number 17/WA/0194) with sponsorship provided by Swansea Bay University Health Board. The study was performed in accordance with Good Clinical Practice. Pregnant women were recruited via study posters at antenatal clinics in Singleton and Neath Port Talbot Hospitals. Written informed consent was obtained from all participants before the start of the trial. The study protocol has been previously published.^30^

### Participants

Women taking part in the trial were required to be at least 18 years of age at 16–18 weeks’ gestation with at least one of the following risk factors for GDM: BMI >30 kg/m^2^, previous macrosomic baby (>4.5 kg), previous episode of GDM or a family history of type 2 diabetes mellitus (first degree relative). Participants were excluded if they were unable to or unwilling to sign informed consent, had previous diabetes mellitus or were on treatment with metformin, had known chronic infection, for example, hepatitis, HIV, had chronic kidney, liver or heart disease or had received previous bariatric surgery.

### Study visits

Participants (n=83) were seen on two occasions at 16–18 weeks gestation and 24–28 weeks gestation. At both visits, demographic data (age, weight, height, BMI, week of gestation) were recorded by clinical research nursing staff. At visit 1, participants underwent a 75-g OGTT to determine their glycaemic status according to NICE diabetes in pregnancy guidelines.^2^ The women were classified as either normal glucose tolerant (NGT) or glucose intolerant (reaching the threshold for GDM). Women who reached the GDM threshold (n=12) were excluded from the study as at 16–18 weeks gestation, it is unknown if the glucose intolerance detected was pre-existing before conception. They were referred back to the antenatal clinic and followed with standard diabetes in pregnancy care.

**Figure 1:**
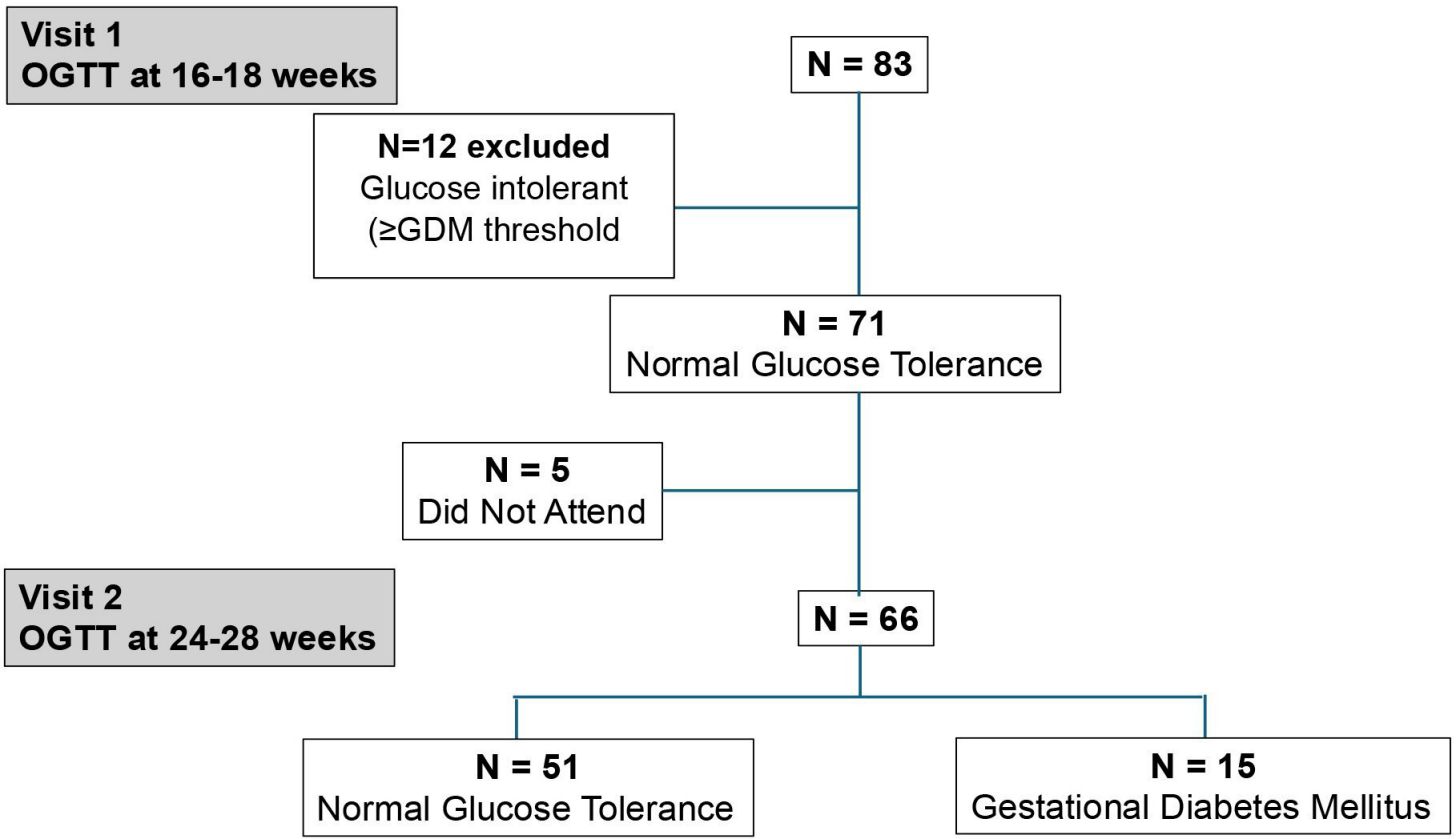
Enrolment of study participants.

In the participants who remained in the study, at visit 2 (24–28 weeks), another 75 g OGTT was performed and the participants (n=71) were categorised as either NGT or GDM (figure 1).

### OGTT procedure and sample collection

Following an 8-hour overnight fast, all participants underwent a 75-g OGTT (Polycal, N.V. Nutricia, Trowbridge, UK). Approximately 4 mL of blood was drawn into both an Ethylenediaminetetraacetic acid (EDTA) blood tube and a fluoride oxalate blood tube (Becton Dickinson, Oxford, UK) at fasting, 30, 60 and 120 min post-glucose load. Samples were transported immediately to the laboratory as whole blood, inverted and mixed thoroughly before the sample was centrifuged at 2000 g for 5 min. The plasma glucose concentration was immediately analysed from the fluoride oxalate blood tube, while plasma from the EDTA blood tubes was aliquoted and frozen at −20°C immediately until analysis for total proinsulin, intact proinsulin, insulin and C-peptide. Glucose samples were measured using a glucose oxidase assay (YSI 2300 Stat plus, Fleet, Hants, UK). Total and intact proinsulin, insulin and C-peptide were measured using specific immunoassays using chemiluminescent labels (IV2-003, IV2-002, IV2-001 and IV2-004; Invitron Ltd, Monmouth, UK).

### Data analysis

Women who were glucose intolerant (reaching the threshold for GDM) at visit 1 (16–18 weeks) were excluded from further participation in the study (n=12) but their demographic and glucose data are presented in table 2. Those who remained non-diabetic throughout are labelled ‘NGT’ and those that were classified as GDM at visit 2 (24–28) weeks despite being NGT at 16–18 weeks are labelled ‘Progressors’.

All analysis and data preparation were performed using the statistical software SPSS Version 22. Values were checked for normality. Data are displayed as mean (SD) for parametric data. Statistical hypothesis tests were performed at 5% significance level. Direct comparisons between groups at each study visit were assessed using independent sample t tests.

Receiver operator characteristic (ROC) analysis was performed using week 16–18-week data to identify any analytes/time points that would discriminate between participants who remained NGT and those who progressed to GDM at 24–28 weeks (AUC p<0.05).

### Patient and public involvement

In preparation for this study, the Public Reference Group of the Diabetes Research Unit Cymru was consulted and their opinions sought on the concept of the study and the study design.

## Results

Participant enrolment is represented in figure 1. The original intention was to enrol a sample size of n=200 based on the ability to estimate with precision of ±0.06 for an expected sensitivity of 0.9 with the proinsulin tests at the significance level of 0.05. The prevalence of GDM in this high-risk group was assumed at 30% for power calculations.^30^ Due to the COVID-19 pandemic, recruitment for the study had to be stopped as the maternity unit adopted different screening tests. Analysis of the data has therefore been carried out on a reduced sample size.

A total of 83 patients were recruited to the study before enrolment had to be suspended due to the pandemic. Of these 12 reached the threshold for GDM at the first visit and were removed from the study and referred back to the clinicians in the antenatal clinics. 71 patients continued with the study, of which 5 did not attend visit 2. The remaining 66 patients underwent the second OGTT, of which 51 remained normal glucose tolerant while 15 developed GDM. Baseline demographic data of these groups are presented in table 1.

**Table 1:** Mean (SD) baseline demographic data of the study participants

	Glucose intolerant (≥GDM threshold) at visit 1	Remained normal glucose tolerant at visit 2 (‘NGT’)	GDM at visit 2 (‘Progressors’)
N	12	51	15
Age (years)	31.2 (5.4)	29.8 (5.8)	34.3 (4.9)*
Weight (kg)	98.8 (27.6)	95.1 (16.6)	94.7 (16.3)
Height (cm)	165.8 (9.9)	165.6 (6.4)	166.7 (8.1)
BMI (kg/m^2^)	35.5 (7.3)	34.6 (5.2)	34.3 (6.3)
Week of gestation at visit 1	16.7 (0.49)	16.9 (0.72)	17.1 (0.70)
Week of gestation at visit 2	N/A	27.9 (0.42)	28.1 (0.62)

*P<0.05 compared with NGT at visit 1 (independent samples t test).

BMI, Body Mass Index; GDM, gestational diabetes mellitus; N/A, not applicable; NGT, normal glucose tolerant.

At visit 1, demographic data were similar between the patients that remained NGT and those that progressed to GDM at visit 2, with the exception that those who developed GDM were older (NGT: 29.8 (5.8) years; Progressors 34.3 (4.9) years; p<0.05).

**Figure 2:**
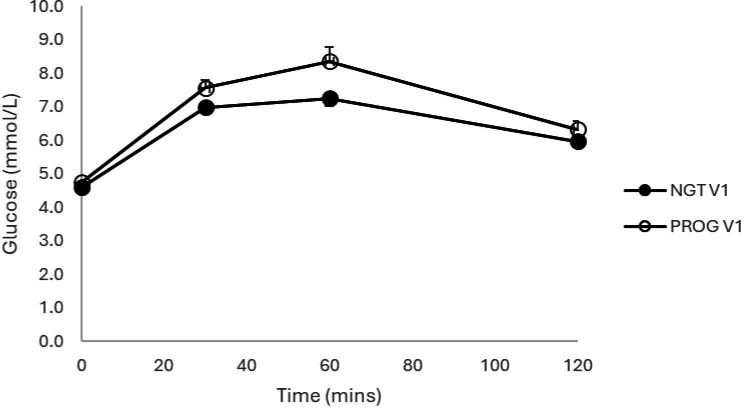
Plasma glucose levels (mean±SEM) at first visit comparing patients progressing to GDM to those that remained normal glucose tolerant. GDM, gestational diabetes mellitus.

Comparison plasma glucose concentrations are presented in figure 2. There were no differences observed between fasting (4.60±0.04 vs 4.75±0.08 mmol/L), 30 min (7.00±0.16 vs 7.57±0.24 mmol/L) and 120 min (5.97±0.13 vs 6.33±0.25 mmol/L) glucose values (all p<0.05). However, the 60-min glucose was significantly greater in those progressing to GDM (7.26±0.22 vs 8.36±0.43 mmol/L; p=0.03).

**Figure 3:**
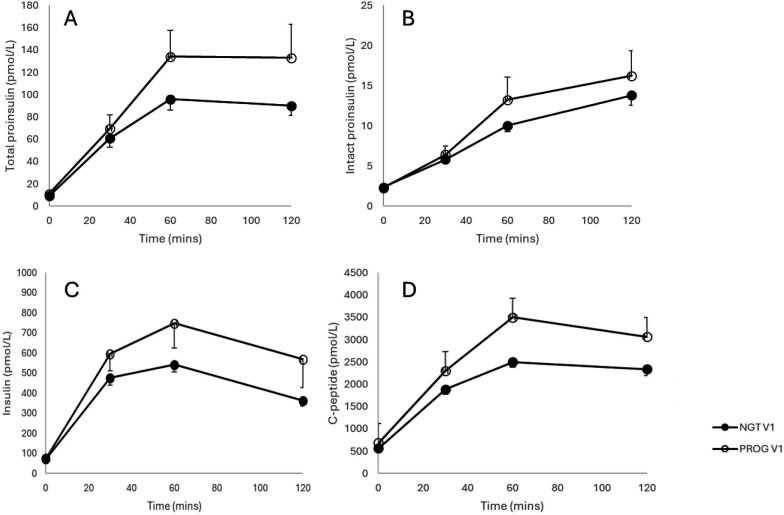
Pancreatic beta-cell profiles (mean±SEM) at first visit comparing patients progressing to GDM to those that remained glucose tolerant. A=total proinsulin, B=intact proinsulin, C=insulin, D=C-peptide. GDM, gestational diabetes mellitus.

Figure 3 illustrates plasma total and intact proinsulin, insulin and C-peptide profiles in response to the oral glucose load in the two groups of patients.

**Figure 4:**
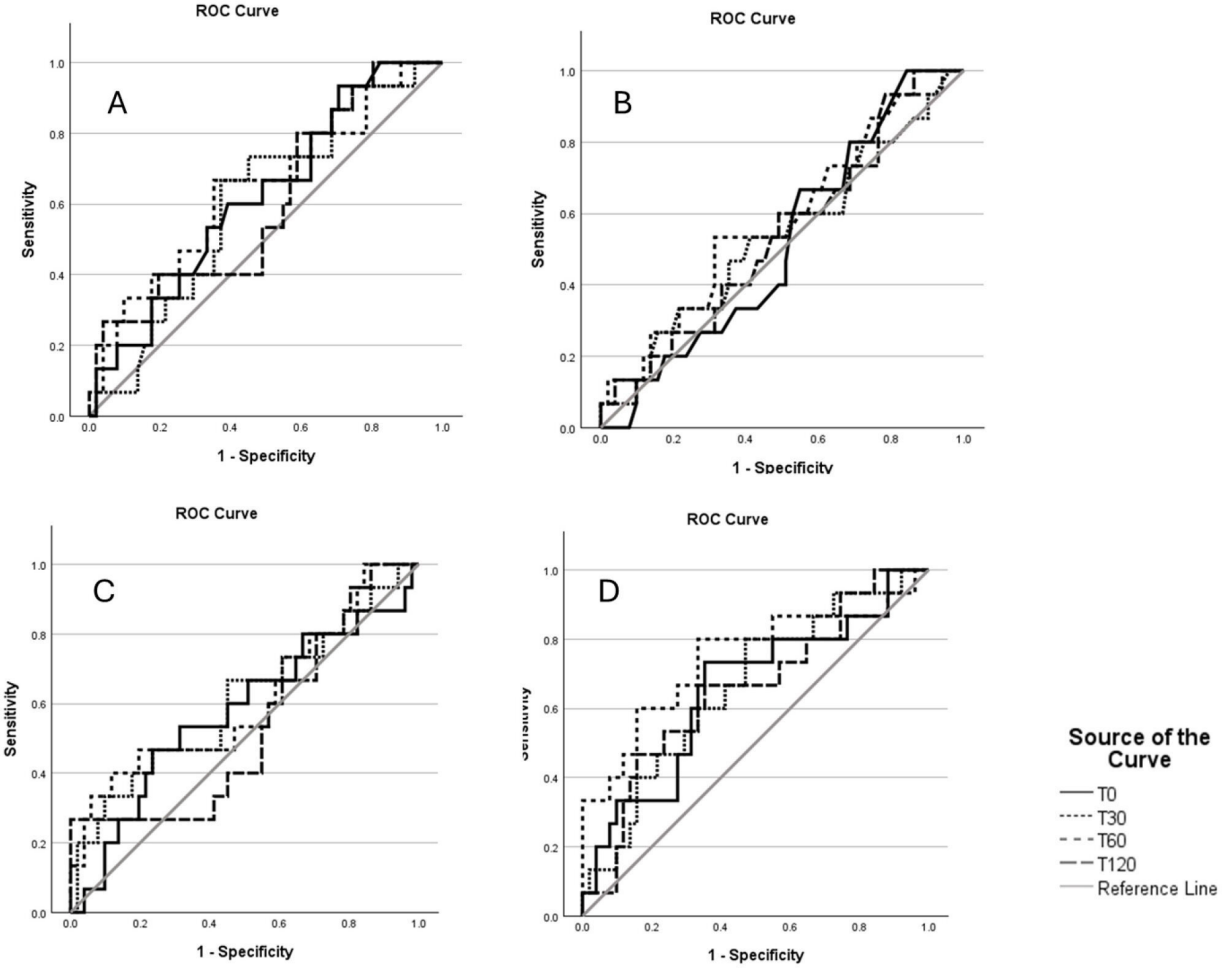
Pancreatic beta-cell products receiver operator characteristics (A=total proinsulin, B=intact proinsulin, C=insulin, D=C-peptide).

Figure 4 shows the receiver operator characteristic curves for plasma total and intact proinsulin, insulin and C-peptide.

Receiver operator curve AUCs for visit 1 plasma total and intact proinsulin, insulin and C-peptide measurements are shown in table 2.

**Table 2:** Receiver operator characteristics area under the curve derived from visit 1 measurements

	Time (min)	AUC	SE	P values
Total proinsulin	0	0.620	0.079	0.126
	30	0.603	0.079	0.191
	60	0.641	0.084	0.094
	120	0.603	0.083	0.218
Intact proinsulin	0	0.517	0.078	0.828
	30	0.537	0.088	0.676
	60	0.578	0.085	0.361
	120	0.542	0.083	0.615
Insulin	0	0.575	0.089	0.399
	30	0.607	0.089	0.233
	60	0.609	0.090	0.228
	120	0.533	0.089	0.708
C-peptide	0	0.654	0.084	0.066
	30	0.661	0.079	0.041
	60	0.744	0.083	0.003
	120	0.654	0.082	0.060

AUC, area under the curve.

There were no significant differences between ROC AUCs and 0.5 at any time points for total proinsulin, intact proinsulin and insulin. However, at visit 1 (16 to 18 weeks), 30 and 60 min C-peptide concentrations were able to discriminate between patients who remained NGT and patients who progressed to GDM at 24–28 weeks, with significant differences observed between ROC AUC and 0.5 (30 minute AUC 0.661±0.079; p=0.041 and 60 min AUC 0.744±0.083; p=0.003) following a standard 75 g oral glucose load.

## Discussion

GDM occurs in up to 10% of pregnancies in the western world.^31^ NICE recommends targeted screening for GDM at 24–28 weeks in the high-risk group to help manage scarce resources. In our cohort, the prevalence of GDM in this high-risk group was 32.5%. Moreover, of these, 15% of women were diagnosed at the first visit (16–18 weeks), and if NICE guidelines had been followed, these women would have continued to be hyperglycaemic until their routine OGTT at 24 weeks.

A recent systematic review and meta-analysis^32^ has indicated no good evidence for any of the diagnostic criteria for early-onset GDM with a suggestion to use fasting glucose of 6.1–6.9 mmol/L in the first trimester. Others have tried to develop prediction models for obese women at high risk of GDM.^10^ Mittendorfer *et al* found that following an OGTT, both 60 min glucose was higher and total glucose AUC 0–120 min was higher in overweight or obese women who went on to develop GDM at 15 weeks gestation.^33^

Early diagnosis of GDM therefore still remains a problem, and insulin and its precursors, including proinsulin, have previously shown promise. A study by Clark *et al* in 1997 in 179 women (52 who developed GDM and 127 who did not) who had positive 1 hour screening tests at between 16 and 33 weeks gestation demonstrated that a combination of fasting and 2-hour insulin levels, fasting free fatty acids and fasting β-hydroxybutyrate were all statistically significant individually and jointly in terms of predictive value lending to the argument that GDM is an insulin-resistant state.^34^ Another study by Bito *et al* in 2005 found that first trimester fasting and 2-hour insulin levels were predictive for development of GDM.^35^ Fasting 2-hour insulin levels had a sensitivity and specificity of 69.2% and 96.4% respectively, and 2-hour insulin levels had a sensitivity and specificity of 92.3% and 85.7%, respectively.^35^ A study by Genova *et al* confirmed elevated proinsulin levels at 24–28 weeks in 53 women with GDM and 49 women without GDM^27^ while Swinn *et al* also found elevated levels of proinsulin in those who had GDM and also found it was strongly associated with subsequent GDM development although these measurements in these studies were carried out at 28 to 32 weeks’ gestation.^28^ In contrast, our study has not found an association between the risk of development of GDM and proinsulin or insulin concentrations in early pregnancy. Our results indicate that while there was some evidence of beta-cell dysfunction (increased proinsulin concentrations) at 16 weeks in the women who progressed to GDM compared with those with NGT, there was no statistical difference. This could well be due to the small numbers and a larger cohort may have demonstrated different results as although our study was powered to recruit 200 pregnant women, unfortunately, recruitment had to be stopped due to the COVID-19 pandemic. This was a major limitation of the study. It was also observed that the concentrations of intact proinsulin were much lower compared with concentrations observed in a non-diabetic, non-pregnant general population. Similar low concentrations of proinsulin have been reported previously and it has been suggested that enhanced efficiency of proinsulin processing in response to insulin resistance in pregnancy may be contributing.^36^

In contrast, statistically increased concentrations of visit 1 C-peptide at 30 and 60 min (ROC AUC 0.661±SE 0.079, p=0.041; 0.744±SE 0.083, p=0.003 respectively) following the OGTT were observed, discriminating between those who remained NGT and those progressing to GDM at visit 2. Elevated concentrations of C-peptide in early pregnancy in women who will go on to develop GDM have also been reported in the study by Clark *et al*.^34^ Elevated levels of C-peptide were detected during OGTT at fasted (p=0.002) and 2-hour (p=<0.001) time points. Furthermore, another study of 996 pregnant women in China found that elevated C-peptide in early pregnancy was associated with a higher risk of GDM (OR [95% CI] 2.28 (1.43 to 3.62).^37^ This study looked at a predictive performance model using multiple variables including C-peptide at 11 weeks gestational age. This cohort of patients was also non-obese with prepregnancy BMI 21 kg/m^2^. These findings are surprising given the very early gestation and the lean cohort of patients. Our Caucasian cohort is more representative of women seen in clinics locally, notwithstanding the much smaller numbers. In contrast, a study by Mittendorfer *et al* found elevated C-peptide in women with GDM at 35 weeks gestation but found no predictive value in levels of C-peptide at 15 weeks gestation during OGTT.^33^

Strengths of our study include its prospective, longitudinal nature with all participants recruited and followed up at a single site. Additionally, the hormone data presented were all generated using sensitive and highly specific chemiluminescent immunoassays, avoiding the potential significant cross-reactivity often observed with previous assays. The major limitation of the study was that the COVID-19 pandemic necessitated an early study termination resulting in a lower than planned for sample size; thus, the study was underpowered and may have under-reported potential significant findings.

Our findings confirm the potential for measurement of C-peptide at 16–18 weeks in high-risk pregnant women to identify those who are likely to go on to develop GDM. Larger studies with suitable power would need to be done to validate these findings.

## Data Availability

Data are available upon reasonable request.
