# Supplementary material for: Pancreatic beta-cell secretory products in the diagnosis and risk stratification of gestational diabetes mellitus: a prospective longitudinal cohort study
Source: BMJ Open. 2025 Sep 5;15(9):e100039. doi: 10.1136/bmjopen-2025-100039 (PMC12414196; doi:10.1136/bmjopen-2025-100039)
Supplement: Supplementary file 1 [file bmjopen-2025-100039.reviewer_comments.pdf]

PEER REVIEW HISTORY

BMJ Open publishes all reviews undertaken for accepted manuscripts. Reviewers are asked to complete a checklist review form and are provided with free text boxes to elaborate on their assessment. These free text comments are reproduced below.

ARTICLE DETAILS

Title (Provisional)

Pancreatic Beta-cell Secretory Products in the Diagnosis and Risk Stratification of Gestational Diabetes Mellitus: a prospective longitudinal cohort study

Authors

Dunseath, Gareth; Atkinson, Michael; Cheung, Wai-Yee; Luzio, Stephen; Peter, Rajesh

---

VERSION 1 - REVIEW

**Reviewer**	**1**
**Name**	**Gunasekaran, Uma**
**Affiliation**	**University of Texas Southwestern Department of Internal Medicine, Division of Endocrinology and Metabolism**
**Date**	**27-Feb-2025**
**COI**	**None**

This is an important topic to explore and this study was well-constructed, but the low enrollment compared to the anticipated sample size makes this study difficult to interpret.

There has been a lot of research done in this area to identify factors that can help identify pregnant women earlier in pregnancy to identify GDM. There is established comorbidity associated with the diagnosis both for the mother and the fetus. That being said, there has been a lot of difficulty in identifying tests that can reliably identify these women earlier as the authors have also presented.

This predictive model study (Yang, X., Ye, Y., Wang, Y. et al. Association between early-pregnancy serum C-peptide and risk of gestational diabetes mellitus: a nested case–control study among Chinese women. Nutr Metab (Lond) 19, 56 (2022). https://doi.org/10.1186/s12986-022-00691-3) identifies c-peptide as a possible early marker. Other studies only look at c-peptide in fasting samples earlier in pregnancy. This is the first study that is measuring c-peptide and other markers during an OGTT. This is not clearly stated in the discussion as to why this study is therefore important even with the smaller sample size. This needs to be re-written to emphasize this.

Line 7 (p.4): carbohydrate intolerance- this can include maldigestion of carbohydrates; perhaps use insulin resistance? unclear what this means

---

**Reviewer**	**2**
**Name**	**Mason, Deidre**
**Affiliation**	**Tygerberg Hosp, Obstetrics and Gynaecology**
**Date**	**27-Feb-2025**
**COI**	**None**

Thank you for the opportunity to review this manuscript of your important work. The article is well-written, reads easily with logical flow. I want to highlight a few concerns.

The submitted manuscript presents a prospective, longitudinal cohort study where pregnant persons, in the context of risk-based selective gestational diabetes screening (GDM) were offered oral glucose tolerance tests (75g OGTTs) at two time points (16-18 weeks and 24-28 weeks). At the early visit, samples were analysed for glucose, total and intact proinsulin, insulin and C-peptide to explore the utility of these markers to predict GDM diagnosis at the second visit.

Title and also Abstract (introduction) pg 2 lines 28-29

"an early marker to diagnose and assess risk of developing GDM"

The beta-cell secretory products were interrogated for validity to predict GDM (diagnosed by 'gold-standard' 75g OGTT at 24-28w). Can the authors motivate why they include the biomarkers having been explored as diagnostic?

The introduction reads well and the various biomarkers that have been explored in this context are mentioned. However, when discussing the work done up to now on proinsulin, the studies quoted are notably old (six published in the 1990s). On a brief search, I also did not uncover more recent published work in this domain. Do the authors have a theory about why there is this long gap before proinsulin is re-explored? Should the fact that the previous studies quoted on pg 6 line 20-27 are so outdated, be commented on?

pg 7 line 35 - I believe this is the first use of the abbreviation 'NGT' . Please check this is used in full, prior to the abbreviation.

pg 8 line 24-26

Women found to be 'glucose intolerant' were excluded at 16-18weeks. "Glucose intolerant" is not really a term used in modern diabetes in pregnancy management. Currently, in practice, we differentiate early GDM and 'overt diabetes' based on the severity of the hyperglycaemia. This group used NICE criteria for GDM diagnosis (5.6mmol/l / 7.8mmol/l) but in the excluded group don't indicate the level of hyperglycemia (where fasting 7.0mmol/l or more or 2hr value of 11.1mmol/l or more would be considered overt criteria). This may

not be relevant as the women were excluded from the study, HOWEVER, they are later included when the prevalence is quoted as 32.5% (pg 11 line 38). If the data showing the extend of the hyperglycaemia at the first visit (in the excluded cohort of participants) is not given/discussed, it would be difficult to justify including them in the denominator as "GDM".

My main concern with the study is the impact of the sample size.

Power calculations, using a prevalence of 30% GDM In the high-risk (screened) group, advised a sample size of 200.

Due to limitations, the sample size recruited was 83 of which only 66 were included at the second visit. If the 12 participants excluded at the first visit due to hyperglycaemia are excluded, the prevalence is much lower (~22%).

Even if the AUC appears good for some markers, the small sample size and low incidence makes generalisation to a larger population a significant concern. The authors acknowledge the small sample size in the limitations.

A voice that I'm missing is the potential clinical utility of testing biomarkers during an early OGTT. How would this translate to benefit patient care? Would the authors advise, for example, medical nutrition therapy to be introduced in those who screen high risk at 16-18 weeks?

I believe there is merit in this work and that it will contribute to the theoretical body of knowledge and could be considered for publication with some modifications and consideration of the statistical implications especially.

**Reviewer**	**3**
**Name**	**Wiegand, Martin**
**Affiliation**	**UCL, Biostatistics Unit**
**Date**	**14-Apr-2025**
**COI**	**I have not, but the webpage didn't let me proceed without entering something**

The authors report the findings of a study on biomarkers collected during OGTT in pregnant women at risk of gestational diabetes (GDM), and how these markers could be early predictors for GDM. Unfortunately this study had to stop recruitment considerably early, due to the onset of the COVID-19 pandemic, which meant that the original aims of the project could not be carried out as planned, and this analysis was performed instead with the sample that could be collected.

Unfortunately the authors could not find a lot of significant relationships, which was also attributed to the insufficient sample size. However, there are some key issues that need resolving before I would recommend publication. Most of these centre around how the pivot

in research topic was documented in the paper, and the structure of the statistical considerations. The shift in research strategy needs to be properly reasoned, and suitable methodology used. The main points are outlined below, with additional notes left in a commented version of the script.

I recommend a major revision, with either detailed justification of the current methodology, or an extension to make more efficient use of the data that is available.

Strength and weaknesses should acknowledge that the initial aim of the project changed due to the sample size necessary for the planned project was not met. This should also be reiterated in the methodology section

The sample size was calculated based on estimating the precision of proinsulin tests. However, there is no analysis relevant to this sample size calculation. I presume this is due to the limited sample size forcing a change in the analysis plan, but this needs to be stated clearly.

The univariate analyses of the biomarkers to predict GDM progression does not account for any potential confounders. The baseline table itself acknowledged that some baseline characteristics were not evenly distributed between recruitment groups.

If the ability to predict GDM based on the biomarkers collect during the OGTT, then why was the next step not to develop a model that accounts for other risk factors and includes multiple of the collected hormones? A variable selection could then be conducted to build an efficient model. From logistic regression to random forests a whole band of approaches could have been considered. Perhaps this would have yielded better results than the univariate analysis which yielded almost entirely non-significant results.

As far as I can tell, all measurements at 30 minutes, 1 hour, 2 hours etc after the glucose are used independently. Why not use them together to optimise use of the data? Summary measures for the biomarkers could be used, if keeping to a standard logistic regression model. Or a mixed model, accounting for repeated measurements over time, or possibly functional data analysis? In summary, why look only at individual measurements, if the entire trajectory of repeated measurements could be considered?

**** **The reviewer provided a marked copy with additional comments. Please contact the publisher for full details.** ****

VERSION 1 - AUTHOR RESPONSE

Reviewer: 1

Comments to the Author:

- *This predictive model study (Yang, X., Ye, Y., Wang, Y. et al. Association between early-pregnancy serum C-peptide and risk of gestational diabetes mellitus: a nested case–control study among Chinese women. Nutr Metab (Lond) 19, 56 (2022). https://doi.org/10.1186/s12986-022-00691-3) identifies c-peptide as a possible early marker. Other studies only look at c-peptide in fasting samples earlier in pregnancy. This is the first study that is measuring c-peptide and other markers during an OGTT. This is not clearly stated in the discussion as to why this study is therefore important even with the smaller sample size. This needs to be re-written to emphasize this*.

  The importance and differences of our study compared to the study by Yang X et al has now been included in the Discussion section of the main text.

- *Line 7 (p.4): carbohydrate intolerance- this can include maldigestion of carbohydrates; perhaps use insulin resistance? unclear what this means.*

  The sentence has been amended to make it clearer and the reference has been updated.

Reviewer: 2

Comments to the Author:

- *Title and also Abstract (introduction) pg 2 lines 28-29*
  "an early marker to diagnose and assess risk of developing GDM"
  The beta-cell secretory products were interrogated for validity to predict GDM (diagnosed by 'gold-standard' 75g OGTT at 24-28w). Can the authors motivate why they include the biomarkers having been explored as diagnostic?

  If one looks at the pathophysiology of GDM- insulin resistance starts to rise after the first trimester, requiring pancreatic beta-cells to secrete increased amounts of insulin to maintain euglycaemia. Within the pancreatic beta-cells, insulin processing starts with cleavage of pre-proinsulin to proinsulin and its cleavage intermediates (Intact and total), to finally form insulin and C-peptide. With increased demand for insulin, these precursors of insulin including total and intact proinsulin and also C-peptide are released in increased quantities. This usually precedes hyperglycaemia as increased secretion initially can keep glucose levels in the normal range. This is also explained in our protocol publication of the study (1). This is similar in type 2 diabetes but the process takes longer as increase in insulin resistance with changes in weight happen over a period of time. There have been screening studies looking to evaluate if these very same precursors could be used in the diagnosis of type 2 diabetes mellitus (2).
  1. Peter R, Bright D, Cheung WY et al. Proinsulin in the identification and risk stratification of gestational diabetes mellitus; study protocol for a prospective, longitudinal cohort study. BMJ Open. 2018 Aug 29;(8):e022571
  2. Ward WK, LaCava EC, Paquette TL et al. Disproportionate elevation on immunoreactive proinsulin in type 2 (non-insulin dependent) diabetes mellitus and in experimental insulin resistance. Diabetologia 1987; 30: 698-702

- *The introduction reads well and the various biomarkers that have been explored in this context are mentioned. However, when discussing the work done up to now on proinsulin, the studies quoted are notably old (six published in the 1990s). On a brief search, I also did not uncover more recent published work in this domain. Do the authors have a theory about why there is this long gap before proinsulin is re-explored? Should the fact that the previous studies quoted on pg 6 line 20-27 are so outdated, be commented on?*

  It is difficult to speculate as to why publications relating to proinsulin are noticeably old, and there have been no recent publications in the domain. Assays to specifically measure proinsulin and its conversion intermediates were introduced in the 1980's, however have not been introduced into routine practice, and therefore the lack of routine data may not have attracted the attention of researchers in the field.

- *pg 7 line 35 - I believe this is the first use of the abbreviation 'NGT' . Please check this is used in full, prior to the abbreviation.*

  Normal glucose tolerant (NGT) has been used in full previously- Page 2 line 44

- pg 8 line 24-26 - *Women found to be 'glucose intolerant' were excluded at 16-18weeks. "Glucose intolerant" is not really a term used in modern diabetes in pregnancy management. Currently, in practice, we differentiate early GDM and 'overt diabetes' based on the severity of the hyperglycaemia. This group used NICE criteria for GDM diagnosis (5.6mmol/l / 7.8mmol/l) but in the excluded group don't indicate the level of hyperglycemia (where fasting 7.0mmol/l or more or 2hr value of 11.1mmol/l or more would be considered overt criteria). This may not be relevant as the women were excluded from the study, HOWEVER, they are later included when the prevalence is quoted as 32.5% (pg 11 line 38). If the data showing the extend of the hyperglycaemia at the first visit (in the excluded cohort of participants) is not given/discussed, it would be difficult to justify including them in the denominator as "GDM".*

  The same NICE criteria (5.6mmol/l / 7.8mmol/l) were used to diagnose GDM in both the 'diagnosed' and 'excluded' group. The 32.5% refers to all the patients diagnosed with GDM in the study, as a percentage of the women screened according to the risk factors as specified by NICE.

- *My main concern with the study is the impact of the sample size.*
  Power calculations, using a prevalence of 30% GDM In the high-risk (screened) group, advised a sample size of 200.
  Due to limitations, the sample size recruited was 83 of which only 66 were included at the second visit. If the 12 participants excluded at the first visit due to hyperglycaemia are excluded, the prevalence is much lower (~22%).
  Even if the AUC appears good for some markers, the small sample size and low incidence makes generalisation to a larger population a significant concern. The authors acknowledge the small sample size in the limitations.

As indicated, the small sample size has been acknowledged as a shortcoming of the study.

- *A voice that I'm missing is the potential clinical utility of testing biomarkers during an early OGTT. How would this translate to benefit patient care? Would the authors advise, for example, medical nutrition therapy to be introduced in those who screen high risk at 16-18 weeks?*

  In clinical practice we would advise starting home blood glucose monitoring / continuous glucose monitoring in this high-risk group at 16-18 weeks, and if glycaemia was above target metformin/insulin treatment could be started earlier to potentially reduce the complications associated with GDM. One could speculate that 15% (page 11 line 38) of women who were GDM at the initial visit (and would not normally have had an OGTT performed until 24-28 weeks according to NICE criteria) would have been identified and early management interventions could have started.

Reviewer: 3

Comments to the Author:

- *Please see the file attached for further comments.*
  We have responded to these comments below.

- *I recommend a major revision, with either detailed justification of the current methodology, or an extension to make more efficient use of the data that is available.*

  Due to the pandemic, the study was terminated early. The study team was wary about the risk of potential bias and reduced statistical power due to the smaller than anticipated sample. The team therefore decided to adopt a more simple approach to perform only the basic statistical analysis related to accuracy of diagnostic tests and reported the findings, highlighting the limitations and the care required for interpretating the findings.

- *Strength and weaknesses should acknowledge that the initial aim of the project changed due to the sample size necessary for the planned project was not met. This should also be reiterated in the methodology section*

  Our original primary aim was to “establish” if proinsulin concentrations at 16-18 weeks gestation would help identify or risk stratify high risk pregnant women who go on to develop GDM. With a smaller than anticipated sample size, we acknowledge that our original primary aim was not met. Instead, we are only able to “explore” if proinsulin concentrations at 16-18 weeks gestation would help identify or risk stratify high risk pregnant women who go on to develop GDM. We therefore have amended our aim statement accordingly.

- *The sample size was calculated based on estimating the precision of proinsulin tests. However, there is no analysis relevant to this sample size calculation. I presume this is due to the limited sample size forcing a change in the analysis plan, but this needs to be stated clearly.*

  The sample size was calculated based on the precision of the proinsulin tests. As we were unable to achieve our targeted sample, we were not able to identify a clinically and analytically reliable cut-off value for the tests at this stage with confidence. We therefore, did not report the confidence intervals of the sensitivity and the specificity but reported a composite measure AUC as an indicator of the precision of the proinsulin tests for future research.

- *The univariate analyses of the biomarkers to predict GDM progression does not account for any potential confounders. The baseline table itself acknowledged that some baseline characteristics were not evenly distributed between recruitment groups.*
  If the ability to predict GDM based on the biomarkers collect during the OGTT, then why was the next step not to develop a model that accounts for other risk factors and includes multiple of the collected hormones? A variable selection could then be conducted to build an efficient model. From logistic regression to random forests a whole band of approaches could have been considered. Perhaps this would have yielded better results than the univariate analysis which yielded almost entirely non-significant results.

  We were not confident that the uneven distribution was not due to sampling variation as a result of the early stoppage of the study
  Proinsulin, insulin and C-peptide are strongly inter-related (prohormone and resulting hormones) and could not be included in the same model. Variable selection may be able to help us to identify the biomarkers with the best potential, but we are not confident that the small sample will not lead to risk of model overfit, model instability and mis-interpretation due to potential bias.
  In addition, although constructing a model that included other risk factors and collected hormone profiles may have provided better results, clinically this would not have been very useful. In clinical practice, a single hormonal test measured at a pre-specified gestational age would be most useful in busy ante-natal clinics.

- *As far as I can tell, all measurements at 30 minutes, 1 hour, 2 hours etc after the glucose are used independently. Why not use them together to optimise use of the data? Summary measures for the biomarkers could be used, if keeping to a standard logistic regression model. Or a mixed model, accounting for repeated measurements over time, or possibly functional data analysis? In summary, why look only at individual measurements, if the entire trajectory of repeated measurements could be considered?*

  The individual times points have been used together in a summary measure and AUCs have been presented. ROC AUC values using all timepoints were no better

than the best individual timepoint AUC value- i.e. 1 or more individual timepoints were always better than the overall value for each biomarker. Although, summary measures with different models could have been used, clinically they would not have been that useful and relevant in day to day use in busy ante-natal clinics. Individual timepoints were also studied to establish best cut-off values

Comments within attached file.

- Page 3: *What is 5 DNA?*

  DNA = Did Not Attend. This has now been put in full in the manuscript

- Page 3: *Possibly this is because I lack medical background knowledge, but it is unclear to me what markers are novel. OGTT test around late 2nd/early third trimester are standard care for risk groups, but what markers are collected? C-peptide is the only one being somewhat promising in your results, but was this only collected in your study? In the discussion you say that for more definitive findings additional data is needed, I wonder why this data can't come from standard EHR data, unless those markers are not routinely collected. If they are not routinely collected, wouldn't this be a considerably cost to change policy?*

  As stated, the OGTT is standard care for patients deemed to be at high risk for developing GDM and is normally carried out at 24-28 weeks. Only blood glucose concentrations are measured in routine practice. Measurement of proinsulin and C-peptide was novel to this study, and these biomarkers are not available in EHR data. We have not carried out a full health economic analysis to determine the costs to change policy.

- Page 6: *This could be written clearer, you have built a prediction model for GDM using those covariates? What model was used? There is no mention of a prediction model in the abstract.*

  The prediction model refers to work previously published (Ref 10. White S et al. Early antenatal prediction of gestational diabetes in obese women: development of prediction tools for targeted intervention. PloS one. 2016;11(12): e0167846). The sentence has been rewritten to make clearer that it is a previously published study. We have not developed a prediction model in this study.

- Page 8: *"with at least one of the following"*

  This has now been changed

- Page 8: *Should that not techincally be in the inclusion/ exclusion criteria?*

  In hindsight this could have been included in the protocol approved by the ethics committee.

- Page 9: *Is it reported how which covariate was transformed?*
  None of the data reported was transformed. The text referring to transformation in the methods has been removed.

- Page 10: *I think there should be more context given. What guidance for sample size calculation was used/ what formula? This should be exactly reproducible without further assumptions.*

  A full description of the sample size calculation was included in the protocol paper (Reference 30: 30. Peter R, Bright D, Cheung WY, Luzio SD, Dunseath GJ. Proinsulin in the identification and risk stratification of gestational diabetes mellitus: study protocol for a prospective, longitudinal cohort study. BMJ Open. 2018 Aug 29;8(8):e022571). We have reproduced the full sample size calculation below:

  "Sample size was estimated with Buderer's formula [15] which used prevalence, level of clinically acceptable precision and a hypothesised level of sensitivity and specificity as the parameters for estimating sample size.

  There were no direct estimates of GDM prevalence in the high-risk population. Prevalence of GDM in the Western world was estimated to be ~10%1 and the relative risk of developing GDM in women with one of the risk factors (BMI >30 kg/m2) was 2.74. [16] There are also reports of women with a family history of diabetes having a >6-fold risk of developing gestational diabetes. [17] Therefore, we have estimated the prevalence of GDM in our target population to be ~30%. We took the conservative approach of basing our sample size estimation on hypothesised sensitivity rather than specificity. Since the estimated prevalence was lower than 50%, this approach would give a higher estimate of the number to be included in the study.

  As a meaningful screening test, a sensitivity of 90% for the circulating concentration of total and intact proinsulin at 16–18 weeks gestation for GDM is expected as established by glucose measurements following an OGTT at 24–28 weeks. The minimal clinically acceptable precision for estimate of sensitivity is 6%. We also expect loss to follow-up to be less than 10%.

  Adjusted for possible loss to follow-up, a sample size of 200 will be required to estimate with precision of ±0.06 for an expected sensitivity of 0.9 with the proinsulin test, at the significance level of 0.05, if the prevalence of GDM is not lower than 30%."

  A reference to this protocol paper has now been included in this section.

- Page 10: *Assumed*

  We have now changed 'taken' to 'assumed'.

- Page 10: *So what I struggle with here is, where the actual hypothesis test/ estimation for which the sample size was calculated is performed? I understand due to C19 there were recruitment issues and the data fell short of what was planned – however*

The original sample size of n=200 was based on anticipated differences in proinsulin concentrations between patients with NGT or GDM. We have acknowledged that this sample size was not achieved and that the study is underpowered. As such we do not want to over interpret any results that we obtained and have treated them as exploratory data.

- Page 10: *So what is with the significant difference in Age? Does that change anything? Could that not be a confounder in your analysis? These are univariate analyses, so they are not adjusted for anything.*

  The only baseline characteristic that was significant between groups was the age. The more important clinical variables including weight, BMI and gestational week at visits were not significantly different between groups. As univariate analysis was used adjustment was not carried out. These would be taken into account as covariates of interest when designing future studies.

- Page 11: *ROC AUC are usually a performance metric for binary classification models, how is this used here? At least to me it isn't obvious what is being done here*

  Receiver operating characteristic (ROC) plots for proinsulin, insulin and C-peptide at 16–18 weeks gestation were established by plotting the proportion of true positives against the proportion of false positives, in order to assess the diagnostic accuracy of developing GDM at 24-2 weeks gestation.

- Page 11*: Ah, so you are using the biomarkers to distinguish between NGT and GDM patients?*

  Yes

- Page 12: *Not sure why this refers to a significant difference, this would rather mean that C-peptide as a classifier performed significantly better than chance.*

  That is correct. C-peptide as a classifier of GDM at 24 to 28 weeks gestation performed significantly better that chance at 30 minutes (p=0.041) and 60 minutes (p=0.003). These p values have been included in the sentence to justify the use of the phrase ‘significant difference’.

- Page 13: *Well your study wasn't powered to find a difference*

  We have acknowledged the lack of power of the study throughout, and have therefore treated the data as exploratory.

- Page 13: *Exactly*

  We agree and have acknowledged the small numbers.

- Page 13: *Statistically significant? What test? Relative to what? This is a larger issue throughout the manuscript, statistical nomenclature is not used properly, and it is often unclear what comparison is referred to, using what test or model.*

  We believe we have used statistical nomenclature correctly, stating that there is statistical significance when p values were <0.05.

VERSION 2 - REVIEW

**Reviewer**	**2**
**Name**	**Mason, Deidre**
**Affiliation**	**Tygerberg Hosp, Obstetrics and Gynaecology**
**Date**	**26-Jun-2025**
**COI**	

Thank you for the opportunity to review the next draft of your manuscript "Pancreatic Beta-cell Secretory Products in the Diagnosis and Risk Stratification of Gestational Diabetes Mellitus: a prospective longitudinal cohort study".

I believe your comments and changes have sufficiently addressed most of my concerns. I would like to reiterate one of my concerns: Using the term 'glucose intolerant' for the group excluded is, in my opinion, too vague. Did they meet NICE criteria for GDM only, or did they meet Overt diabetes criteria? This is a technical point though and probably irrelevant to the findings of the study.

**Reviewer**	**4**
**Name**	**Dack, Kyle**
**Affiliation**	**University of Bristol, MRC Integrative Epidemiology Unit**
**Date**	**14-Jul-2025**
**COI**	**None**

Statistical review summary:

My primary concerns are regarding the reporting of the statistical methodology, and how results are communicated. The statistical analysis section does not describe the statistical tests performed, which limits the replicability and my ability to evaluate or understand what was done. Regarding the results, I have made several suggestions to improve clarity for readers, and some small tweaks would help a lot.

The introduction and discussion are well written and I have no major suggestions. Overall the analysis seems reasonable but would benefit from some more details being added when describing or reporting it.

Abstract:

1. I suggest clarifying in the final sentence of the methodology subheading how this comparison was performed – i.e. the statistical test or model used.

2. I have a few concerns regarding the final two sentences of the results section.

"There were no significant differences at each time point and ROC AUC curves between the two groups for total and intact proinsulin and insulin". Is the first "and" meant to be "in"? If not, the meaning is unclear to me and I suggest rephrasing.

"ROC AUC curves" does not require "curves", because it is the final C in AUC. However, this acronym has not been defined. I suggest adding the definition, or use an equivalent plain English phrase such as predictive accuracy.

It is reported that there are significant differences in the AUC of C-peptide, but it is not stated which outcome the AUC relates to (i.e. what is being predicted).

Introduction

3. Very clear and well written – no suggestions.

Methods:

4. The study dates should be reported possibly in the first paragraph.

5. The methods describe clearly the outcome, and the OGTT procedure. But demographic measurements are also presented in the results, and these should be listed in the methodology, explaining when and how they were measured.

6. The statistical analysis section is minimal and impairs the transparency or replicability of the study. All comparisons performed in the results should be described, including an explanation of the purpose, and the technical details such as the models used, results extracted, and how the results would be interpreted.

Results

7. Table 1. The column labelling could be clearer by replacing V1 with "visit 1", and NGT with "remained non-diabetic" or similar.

8. Table 1. A p-value is reported in the footnote, but the statistical test is also needed. I also suggest adding to the table label that this is data collected at visit 1 (this is mentioned in-text).

9. Table 2. ROC results are reported for various biomarkers, which test the ability to discriminate between two outcomes. I imagine the outcome being tested is GDM diagnosis

vs no diagnosis at visit 2, but this should be directly stated when ROC / AUC is mentioned to avoid readers misinterpreting the findings.

10. Table 2. The table label should report that these measurements were taken in visit 1.

Discussion

11. The results are interpreted fairly, although again please ensure that AUC is reported in relation to a specific outcome. However, a strengths and limitations section is missing, to elaborate on the bullet points reported at the beginning of the paper.

VERSION 2 - AUTHOR RESPONSE

Reviewer: 2
Dr. Deidre Mason, Tygerberg Hosp
Comments to the Author:
Thank you for the opportunity to review the next draft of your manuscript "Pancreatic Beta-cell Secretory Products in the Diagnosis and Risk Stratification of Gestational Diabetes Mellitus: a prospective longitudinal cohort study".

I believe your comments and changes have sufficiently addressed most of my concerns. I would like to reiterate one of my concerns: Using the term 'glucose intolerant' for the group excluded is, in my opinion, too vague. Did they meet NICE criteria for GDM only, or did they meet Overt diabetes criteria? This is a technical point though and probably irrelevant to the findings of the study.

The wording throughout the text/Table 1/Figure 1 has now been updated to clarify that ‘glucose intolerant’ at visit 1 means those reaching the threshold for GDM.

Reviewer: 4
Dr. Kyle Dack, University of Bristol
Comments to the Author:
Statistical review summary:
My primary concerns are regarding the reporting of the statistical methodology, and how results are communicated. The statistical analysis section does not describe the statistical tests performed, which limits the replicability and my ability to evaluate or understand what was done. Regarding the results, I have made several suggestions to improve clarity for readers, and some small tweaks would help a lot.
The introduction and discussion are well written and I have no major suggestions. Overall the analysis seems reasonable but would benefit from some more details being added when describing or reporting it.

Abstract:

1. I suggest clarifying in the final sentence of the methodology subheading how this comparison was performed – i.e. the statistical test or model used.

Extra text has now been included to highlight the ROC AUC performed.

2. I have a few concerns regarding the final two sentences of the results section.
"There were no significant differences at each time point and ROC AUC curves between the two groups for total and intact proinsulin and insulin". Is the first "and" meant to be "in"? If not, the meaning is unclear to me and I suggest rephrasing.

This was an error, the word 'and' has now been changed to 'in' as suggested.

"ROC AUC curves" does not require "curves", because it is the final C in AUC. However, this acronym has not been defined. I suggest adding the definition, or use an equivalent plain English phrase such as predictive accuracy.

ROC and AUC have both been defined in full in this sentence. The word 'curves' after AUC has been removed

It is reported that there are significant differences in the AUC of C-peptide, but it is not stated which outcome the AUC relates to (i.e. what is being predicted).

Text has been added to clarify that significance is between the AUC value and AUC 0.5 (i.e. no discrimination). Significant timepoints were able to discriminate between those who remained NGT and those who progressed to GDM at weeks 24 – 28.

Introduction
3. Very clear and well written – no suggestions.

Methods:
4. The study dates should be reported possibly in the first paragraph.

The text 'carried out between November 2017 and January 2020' has been added to the first sentence of the methods.

5. The methods describe clearly the outcome, and the OGTT procedure. But demographic measurements are also presented in the results, and these should be listed in the methodology, explaining when and how they were measured.

A list of the demographic data collected is now included in the 'study visits' section of the methods, stating that these data were collected at both study visits.

6. The statistical analysis section is minimal and impairs the transparency or replicability of the study. All comparisons performed in the results should be described, including an

explanation of the purpose, and the technical details such as the models used, results extracted, and how the results would be interpreted.

Additional text has now been included in the 'Data analysis' section of the methods.

Results

7. Table 1. The column labelling could be clearer by replacing V1 with "visit 1", and NGT with "remained non-diabetic" or similar.

The column labelling has been updated ('V1' is now 'Visit 1', 'NGT' is now 'remained normal glucose tolerant')

8. Table 1. A p-value is reported in the footnote, but the statistical test is also needed. I also suggest adding to the table label that this is data collected at visit 1 (this is mentioned in-text).

'at Visit 1' and '(independent samples t test)'has been added to the footnote

9. Table 2. ROC results are reported for various biomarkers, which test the ability to discriminate between two outcomes. I imagine the outcome being tested is GDM diagnosis vs no diagnosis at visit 2, but this should be directly stated when ROC / AUC is mentioned to avoid readers misinterpreting the findings.

Extra text has been added to clarify this in the methods, results and discussion sections.

10. Table 2. The table label should report that these measurements were taken in visit 1.

This has now been included.

Discussion

11. The results are interpreted fairly, although again please ensure that AUC is reported in relation to a specific outcome. However, a strengths and limitations section is missing, to elaborate on the bullet points reported at the beginning of the paper.

Text has been included elaborating on the AUC reporting.

A strengths/limitations section has been included at the end of the discussion
